# Relations between mental workload and decision-making in an organizational setting

**DOI:** 10.1186/s41155-017-0061-0

**Published:** 2017-04-03

**Authors:** María Soria-Oliver, Jorge S. López, Fermín Torrano

**Affiliations:** 1 Facultad de Ciencias Jurídicas, Sociales y Humanidades, UNIR-Universidad Internacional de la Rioja, Av. de la Paz, 137, 26006 Logroño, La Rioja, Spain; 2 Facultad de Ciencias Humanas y Sociales, Departamento de Psicología y Pedagogía, Universidad Pública de Navarra, Campus de Arrosadía s/n. Edificio Magnolios, 31006 Pamplona, Spain

**Keywords:** Organizational Setting, Mental Workload, Global Relation, Professional Setting, Subject Dimension

## Abstract

**Background:**

The complexity of current organizations implies a potential overload for workers. For this reason, it is of interest to study the effects that mental workload has on the performance of complex tasks in professional settings.

**Objective:**

The objective of this study is to empirically analyze the relation between the quality of decision-making, on the one hand, and the expected and real mental workload, on the other.

**Methods:**

The study uses an ex post facto prospective design with a sample of 176 professionals from a higher education organization. Expected mental workload (Pre-Task WL) and real mental workload (Post-Task WL) were measured with the unweighted NASA-Task Load Index (NASA-TLX) questionnaire; difference between real WL and expected WL (Differential WL) was also calculated; quality of decision-making was measured by means of the Decision-Making Questionnaire.

**Results:**

General quality of decision-making and Pre-Task WL relation is compatible with an inverted U pattern, with slight variations depending on the specific dimension of decision-making that is considered. There were no verifiable relations between Post-Task WL and decision-making. The subjects whose expected WL matched the real WL showed worse quality in decision-making than subjects with high or low Differential WL.

**Conclusions:**

The relations between mental workload and decision-making reveal a complex pattern, with evidence of nonlinear relations.

## Background

Current organizational settings frequently imply high complexity and high demands from their workers. Occupational activity can therefore involve levels of demands that go beyond the person’s cognitive capacity of analysis and decision-making (Ferrer & Dalmau, 2004; Rolo, Díaz, & Hernández, 2009). The existing empirical studies present indicators along these lines. Thus, recent official surveys in the European setting indicate a growing incidence of labor problems reflected in indicators related to mental demands: level of attention required, rhythm of work imposed, deadlines to be met, and the monotony of the task (INSHT, 2011, 2014).

The level of overload is important for workers’ efficacy and well-being. For this reason, from different areas of knowledge, researchers have attempted to define constructs and instruments that allow its adequate assessment. Among them, the *Mental Workload* is one of the most widely invoked concepts in ergonomic research and practice (Hancock & Meshkati, 1998; Salvendy, 1997; Wickens, 2008; Young, Brookhuis, Wickens, & Hancock, 2015). Mental workload (MWL) attempts to measure the extent to which occupational activity matches or exceeds the worker’s resources. In this sense, it has been studied from two essential perspectives. The first one considers that MWL is a factor that depends exclusively on task demands to which the subject adapts. The second one, currently receiving more support, conceptualizes MWL as a consequence of the relation between task demands and the subject’s skills in terms of a demand/resource balance (Díaz, Rubio, Martín, & Luceño, 2010; Ferrer & Dalmau, 2004; Young et al., 2015). The multidimensionality of the concept of MWL and the subjective perception involved implies different fundamental aspects like characteristics of the task, characteristics of the operator, environmental context in which the performance occurs, time pressure, and subjective aspects related to it, such as stress or the perception fatigue (Hart & Staveland, 1988; Eggemeier, 1988; ISO, 2000). In an attempt to bring these dimensions together and provide a global definition of MWL, Young and Stanton (2005) have suggested that MWL reflects the level of attentional resources required to meet both objective and subjective performance criteria, which may be mediated by task demands, external support, and past experience.

The empirical works carried out have evidenced that MWL presents dynamic and complex relationships with performance, which may also vary according to subjects’ characteristics. In this sense, it has been shown that an increase or decrease of MWL can be compensated to some degree by the investment of additional resources, thus maintaining performance at the cost of individual strain (Hancock & Warm, 2003; Matthews & Davies, 2001). If the intensity and the duration of the effort required to perform the task are balanced, activation will take place in an optimum area, which makes the subject efficient. However, if the effort must be sustained over a prolonged period of time, fatigue appears and the subject’s functional efficiency is temporary altered (ISO, 1991; Pretorius & Cilliers, 2007; Young et al., 2015). On another hand, too little stimulation can lead to underload. In this case, as resources are either allocated elsewhere or otherwise decrease through underuse, the subject’s performance may also be negatively affected (Young & Stanton, 2002; Wilson & Rajan, 1995; Young et al., 2015). Considering the former, as Young et al. (2015) state, there is a strong consensus that mental underload can be just as detrimental to performance as mental overload, with both leading to performance degradation, attentional lapses, and errors. The relationship between MWL and performance may thus follow a similar pattern to the classical “inverted U” of Yerkes-Dodson’s curve (Yerkes & Dodson, 1908), where optimal performance is located between low and high MWL (Brookhuis & Waard, 2002; Young et al., 2015).

However, MWL literature has mainly been centered on measuring performance by means of operative tasks, like those related to traffic or transport research, real flight, flight simulation, air traffic control, peripheral detection, or formal memory/follow-up tasks (see Bowling & Kirkendall, 2012; Brookhuis & Waard, 2002; Hart, 2006; and Young et al., 2015). In this sense, only a few and very recent works have focused on relating MWL and one of the key concepts in skilled work settings: decision-making (Byrne, 2013; Baethge, Müller, & Rigotti, 2016; Jackson, Kleitman, & Aidman, 2014).

The concept of decision-making (DM) differs in relevant characteristics from classical performance indicators used in MWL literature. It entails a sequence of actions that allow understanding how subjects face and solve complex problems in professional contexts (Hodgkinson & Starbuck, 2008). Decisions within professional settings have an interactive social dimension and are based on prior knowledge that, in itself, does not guarantee success when applying the decisions adopted (Argandoña, 2011; Offrage, 2009; Weick & Sutcliffe, 2008). They are, in turn, developed in contexts far from the classical paradigms of rational choice. Thus, decisions are conditioned by elements such as open problems; uncertain and dynamic settings; changing, multiple, and competitive goals; multiple feedbacks; time pressure and contrasts; consequences involving some risk; multiple decision-makers; and external standards (Orasanu & Connolly, 1993; Csaszar & Eggers, 2013). DM has critical effects on professional achievement and acquires special relevance when performance and actions involve direct consequences on people’s integrity or well-being (Secchi, 2014).

As mentioned, evidence about relationships between MWL and DM is scarce. In relation to clinical decision-making, Byrne (2013) has suggested—on the basis of the existing research—that MWL and complexity of the information may be key factors to determine which kind of clinical decision-making is developed: schemata-based methods or conscious metacognition procedures. Baethge et al. (2016) have shown empirically that the influence of MWL on performance quality is moderated by the way in which selection, optimization, and compensation of resources and goals are handled by nurses. Jackson et al. (2014), using driving simulation strategies, have provided support for the existence of a MWL zone of optimal DM performance, below or above which a worse quality of DM is produced, as has been suggested for the relationship between MWL and other classical performance measures. It is also worthwhile to mention the evidence provided by the works that have analyzed the relation between individual DM and stress. Recent reviews and meta-analyses postulate that stress occurs whenever a demand exceeds the regulatory capacity of an organism, particularly in situations that are unpredictable and uncontrollable (Dickerson & Kemeny, 2004; Koolhaas et al., 2011). Stress, in this sense, only refers to situations that are conceptualized within the range of MWL. In addition, the emergence of stress as a function of external demands is mediated by appraisal strategies (Lazarus, 1999). Thus, relations between stress and DM can only be applied to the field of MWL in a limited way. However, it is interesting to briefly mention the existing evidence about the relationship between stress and DM in natural settings. In this sense, stress is thought to relate to dysfunctional strategy use, altered feedback processing, heightened reward sensitivity, and lowered punishment sensitivity (Starcke & Brand, 2012)

According to the former considerations, our research question refers to the way in which MWL is related to DM in organizational settings. There are several reasons that support the relevance of this question, which the present work will address, specifically, the scarcity of works that explore the relation between MWL and DM, the relevance implied in the organizational setting, and the evidence of complex relations between MWL and DM. Our general objective is, therefore, to study in depth the effects of MWL on task performance in the work setting, empirically analyzing the relations between MWL and DM in workers who carry out their activity in real contexts. In this sense, our specific objectives are (1) to analyze the relation between the worker’s expectation of MWL prior to task performance (Pre-Task WL) and the quality of DM after task performance; (2) to analyze the relation between perceived MWL after the task (Post-Task WL) and the quality of the DM performed after the task; and (3) to analyze the relation of differences between the expected MWL and the MWL perceived after the task (differential mental workload), on the one hand, and the quality of the DM carried out, on the other.

To address the concept of MWL, we have drawn from the conceptualization model of the NASA-Task Load Index (NASA-TLX) (Hart & Staveland, 1988). The suitability of this method has been supported by numerous studies because it provides more accurate results than other techniques, such as the SWAT or the modified Cooper-Harper scale (Hill et al., 1992; Rubio, Diaz, Martin, & Puente, 2004). This evidence, along with the simplicity of its use, makes the NASA-TLX currently the most widely used instrument to assess MWL (Noyes & Bruneau, 2007; Rutledge et al., 2009). This method allows rating the task from a multidimensional perspective, so it has been proved useful due to its diagnostic capacity with regard to possible sources of workload (Díaz et al., 2010). Its core strengths include its applicability in a naturalist work setting, as the workers can quickly rate the task carried out both a few moments after its performance and retrospectively (Recarte, Pérez, Conchillo, & Nunes, 2008). In studies carried out on retrospective assessments, high correlations have been found between the data extracted in this way and the immediate scores (Wierwille & Eggemeier, 1993). NASA-TLX measurement procedure is detailed more extensively in the “Methods” section.

In the case of DM, we used the conceptualization and measurement model elaborated by Soria-Oliver and her team (Sanz de Acedo Lizarraga, Sanz de Acedo Baquedano, Soria-Oliver, Closas, 2009; Soria-Oliver, 2010). In this model, DM is conceived as a complex sequence of actions that require attending to different parameters conditioned by the temporal and organizational needs. They are specified in the following steps: planning the process, defining the goals, generating options, evaluating them, and selecting the best by considering both the influence of personal variables and variables of the setting. According to the guidelines of Byrnes (1998), Cannon-Bowers, Salas, and Pruitt (1996) and Cannon-Bowers and Salas (2002), this model assumes three sources of variables that characterize naturalistic decision-making: task, subject, and context variable. Task variables are associated with the nature of the decision itself, for instance, the uncertainty involved in each alternative, pressure of time and available money, quantity and quality of the information, proposed goals, and possible consequences of the decision. Subject or decision-maker characteristics include the performers’ internal factors: motivation, thorough self-regulation of the decision stages, crucial information processing, expertise in a certain domain, and the emotions that almost always accompany a decision. Finally, the environmental characteristics define the context in which the decision takes place, specifically, factors that are not directly a part of the decision task itself: social and work influences and distracting events. This model, in turn, has led to the empirical elaboration of a questionnaire (Decision-Making Questionnaire, DMQ (Soria-Oliver, 2010)) that measures the quality of DM in organizational contexts, considering the different theoretical dimensions of the model. Detailed characteristics of DMQ are presented in the “Methods” section.

Our study draws some tentative hypotheses that are conditioned, in any case, by its exploratory and correlational nature. Thus, we will explore the degree to which the relationship between DM, on the one hand, and Pre-Task WL and Post-Task WL, on the other, follow the general pattern that has been evidenced for other performance indicators (Brookhuis & Waard, 2002; Wilson & Rajan, 1995; Young & Stanton, 2002; Young et al., 2015). This pattern may be defined as follows: first, by a low expected or real perceived MWL range in which low stimulation may decrease the quality of DM strategies; second, by an optimum performance range of expected or real perceived MWL in which balanced stimulation adequately fits subjects’ resources and DM quality is higher; third, a high expected or real MWL range that overwhelms subjects’ resources and may yield worse DM quality. In relation to Differential WL, we expect that expected MWL (Pre-Task WL) may act as a moderator of relationships between Differential WL and DM. When subjects’ expected MWL (Pre-Task WL) is low, real MWL (Post-Task WL) that fits this expectation (Adjusted Differential WL) or remains below it (Low Differential WL) may generate lower DM quality due to absence of stimulation. If subjects with low Pre-Task WL have to cope with higher real MWL than expected (High Differential WL), DM may be better if subjects’ resources are not overwhelmed. When subjects’ expected MWL (Pre-Task WL) is high, lower real MWL (Low Differential WL) would lead to worse DM quality due to stimulation decrease. If real MWL fits expected MWL (Adjusted Differential WL), DM quality will be high, as subjects have adequate stimulation. In this case, if real MWL is higher than expected (High Differential WL), DM may be better only if subjects can manage their resources to cope with the requested context demand. However, our empirical design, which measures professional decisions in a real setting without manipulation, does not allow us to predict a priori which expected or real MWL levels may be found and, consequently, whether they would cover all the different ranges proposed in our hypotheses.

## Methods

Design: ex post facto prospective design, with measurement of the independent variable (MWL) before and after task performance and measurement of the dependent variable (DM) after task performance.

Sample: a total of 175 participants, representative of the professionals belonging to an institution of online higher education. The sample was stratified proportionally to the number of people in the different professional categories in the entire institution (managers, middle managers, professors, and tutors). Participants were selected randomly within each stratus. Inclusion criteria were as follows: having joined the institution more than 3 months ago to ensure adequate familiarity with the tasks, and adequate mastery of the Spanish language. The criteria of a 3-month period was established in accordance with prior consultation with the human resources department about the estimated time to achieve workers’ adequate task performance autonomy, as was also reflected in *Work Position Description* documents. The sample had a mean age of 34.5 years (SD = 7.4) and included 57.8% (*n* = 100) of women. It included managers (2.3%; *n* = 4), middle managers (11.6%; *n* = 20), professors (39.9%; *n* = 69), and tutors (46.2%; *n* = 80). The minimum sample size required to perform the study was estimated at 105 people. Sample size was calculated assuming a 95% confidence level, 80% power, a standard deviation of 60 in both comparison groups, and 10% of losses, to detect a minimum difference of 35 points in the score of the DMQ between the groups with high and low differential workload (see below for a definition).

Measurements: The following variables were measured:

Socio-demographic variables: sex, age, and occupational post within the organization

Decision-making: It was measured by means of the DMQ (Soria-Oliver, 2010). The DMQ has been previously validated in professional settings (Sanz de Acedo Lizarraga et al., 2009). It records the extent to which certain qualified strategies of DM are implemented. Its use yields a global score (DMQ score), scores on 3 scales/factors (Task scale, Subject scale, and Context scale scores) and scores on 10 subscales/sub-factors linked to each one of the factors: (1) Task factor: Uncertainty, Time/Money Pressure, Information and Goals, and Consequences of Decision subscale scores; (2) Subject factor: Motivation, Self-Regulation, Cognition, and Emotion subscale scores; and (3) Context factor: Social Pressure and Work Pressure subscale scores. This questionnaire has consistent internal validity and, in previous studies, has yielded high rates of reliability in the different scales and subscales. Table 1 presents a summary of the meaning of its scales and subscales (Sanz de Acedo Lizarraga et al., 2009).

**Table 1:** DMQ (Decision-Making Questionnaire) scales and subscales

Task factor/scale: Tendency to assess all the aspects related to the scope of the decision task, mainly levels of certainty, information, goals, and consequences of the decision.	
• Uncertainty subscale: measures the degree to which individuals consider their concerns about doubt, risk, and the changes caused by the decision • Time/Money Pressure subscale: determines how individuals organize their activities and their predisposition to compare the results of the decision with the time and money spent • Information and Goals subscale: shows the degree to which individuals attach importance to having adequate data available and defining specific goals to appraise task difficulty • Consequences of Decision subscale: assesses the degree to which subjects assign personal responsibility for the effects of the decision	
Subject factor/scale: Tendency to engage in the process of decision in a motivated, thoughtful, and sincere way, self-regulating the process.	
• Motivation subscale: measures the degree to which individuals launch the decision-making process and maintain interest during the development of its successive phases • Self-Regulation subscale: shows the degree to which subjects tend to plan, monitor, and evaluate results • Cognition subscale: measures the degree to which individuals tend to process information, reason about the steps to be taken, and resolve the difficulties that may emerge during the decision-making process. • Emotion subscale: shows the degree to which individuals create an appropriate mood in order to make the decision	
Context factor/scale: Tendency to discover the influence of the social, community, and occupational environment in the decision process.	
• Social Pressure subscale: shows consideration of the impact on the environment or on other persons when making a decision • Work Pressure subscale: measures the degree to which an individual takes organizational rules and goals into account when making decisions	

Mental workload: It was measured by means of the dimensions considered in the NASA-TLX scale (Hart & Staveland, 1988), which measures workers’ subjective experience of the workload. NASA-TLX uses six dimensions: (1) *Mental demand*: how much mental and perceptual activity was required (e.g., thinking, deciding, calculating, remembering); (2) *Physical demand*: how much physical activity was required (e.g., pushing, pulling, turning, controlling); (3) *Temporal demand*: how much time pressure workers felt was due to the rate or pace at which the task or task elements occurred; (4) *Performance*: how successful was the worker in accomplishing the goals of the tasks; (5) *Effort*: how hard did the worker feel she/he had to work (mentally and physically) to accomplish the level of performance; (6) *Frustration*: how insecure, discouraged, irritated, stressed, and annoyed did the worker feel during the task. Twenty-step bipolar scales are used to obtain ratings for these dimensions. In the classical procedure, a previous weighting phase is performed. This weighting phase requires a paired-comparison task to be performed prior to the workload assessments to combine the six individual scale ratings into a global score. However, recent research has shown the scarce utility of the weighting phase (López, Rubio, & Luceño, 2010). Therefore, we decided to use the subjects’ rating directly on the six dimensions considered in the scale, without carrying out the prior pairwise weighting phase. As pointed out, expected MWL was measured before the task (Pre-Task WL) and perceived MWL was measured after performing the task (Post-Task WL). We also estimated a score that we called differential mental workload, obtained as a result of subtracting the Pre-Task WL score from the Post-Task WL score. In this indicator, negative values imply that the task was less burdensome than expected. Positive values, in contrast, reflect that the subject faced a greater burden than expected. Values approaching zero reflect a match between the expected MWL and the real MWL encountered.

Procedure: Our research team presented the objective and procedure of the study to the Rectorate of the academic organization in which the field work would take place. The Rectorate decided that the proposed research was potentially useful for the organization and also that study procedures followed ethical criteria. Consequently, the workers’ institutional e-mail was provided by the organization. The survey was thus disseminated by means of a link to which access was gained by an e-mail invitation. In the e-mail, we mentioned the nature of the research, the subjects’ voluntary participation, and we guaranteed anonymity of the responses. As an e-mail attachment, subjects received the NASA-TLX questionnaire, which had to be printed to become adequately familiar with the instrument. The way in which participation was designed required the subjects to firstly fill in the adapted paper-and-pencil version of the NASA-TLX questionnaire before beginning a typical task of their job and to complete it again after completing the task. After completing both questionnaires, by means of the web link, access was gained to the pre-task and post-task NASA-TLX questionnaire to enter the data collected on paper. The DMQ was also accessed to allow completing it online directly on screen. A reminder was sent 2 weeks after the original invitation in order to promote participation. Out of a total 250 applications, we obtained 175 completed questionnaires (response rate of 70%).

Analysis: We conducted descriptive and initial exploratory analyses, and we examined the reliability of the scales and subscales. Subsequently, we performed multivariate analysis of variance (MANOVA) test and linear regression to explore the relation between decision-making and the different indicators of mental workload. We used the statistical package IBM® SPSS® Statistics V. 22.0.

## Results

### Psychometric properties and initial exploratory analysis

Table 2 presents, firstly, the descriptive statistics and psychometric results of the different scales and subscales, showing indices of central tendency (means), deviation (standard deviation), and the Cronbach’s alpha index for each scale or subscale. Reliability indicators of the measures of MWL are not included because they represent a cumulative measure of different dimensions that cannot be conceptualized from the approach of a psychological construct measured through indicators. Pearson correlation indices between the different measures of MWL and the different scales and subscales of DM are included.

**Table 2:** Descriptive statistics, reliability, and linear correlation indexes between the variables of the study and the indicators of MWL

	Mean	Standard deviation	Reliability (Cronbach’s *α*)	Pearson correlations		
				Pre-Task Workload	Post-Task Workload	Differential Workload (post-pre)
Pre-Task Workload	80.3	12.3	–	1		
Post-Task Workload	80.8	15.1	–	.63**	1	
Differential Workload	0.17	11.9	–	−.19*	.64**	1
DMQ-Total score	470.9	42.9	.94	.26**	.14	−.01
DMQ-Task	205.3	19.9	.87	.21*	.12	.01
DMQ-Uncertainty	45.8	4.7	.63	.16	.18^*^	.13
DMQ-Time/Money Pressure	61.9	5.8	.71	.14	.10	.05
DMQ-Information and Goals	56.5	7.5	.73	.14	.08	−.01
DMQ-Consequences	41.3	6.5	.60	.13	.03	−.06
DMQ-Subject	177.9	15.8	.83	.31**	.16	−.04
DMQ-Motivation	36.9	3.7	.39	.13	.06	.02
DMQ-Self-Regulation	56.7	6.3	.48	.35**	.18^*^	−.10
DMQ-Cognition	45.2	5.1	.66	.26**	.14	−.01
DMQ-Emotion	37.9	3.9	.45	.20*	.15	.03
DMQ-Context	87.1	8.6	.73	.15	.10	.04
DMQ-Social Pressure	52.0	5.4	.60	.13	.18^*^	.15
DMQ-Work Pressure	35.0	4.4	.57	.16*	.03	−.09

*p < .05; **p < .01;

Regarding the psychometric properties of scales and subscales, we note the excellent properties of the DMQ as a whole and of the Task and Subject scales, with the Context scale providing a more modest indicator. The different subscales yielded disparate results, as some did not reach the level of .6 in the Cronbach’s alpha index; therefore, following the current recommendations (Hogan, 2013), we will not use them separately for the exploratory analysis.

We note, firstly, the existence a high correlation between Pre-Task WL and Post-Task WL. Secondly, we highlight the existence of positive, albeit moderate, correlations between various DMQ scales and subscales and Pre-Task WL, especially in the DMQ dimensions referring to Subject. Also notable, but in the opposite direction, are the absence of linear correlations between Differential WL and the different indicators of DMQ and the low correlations between DMQ and Post-Task WL. We shall now present in detail the results of each one of our analysis objectives.

### Relation between Pre-Task WL and decision-making

**Fig. 1:**
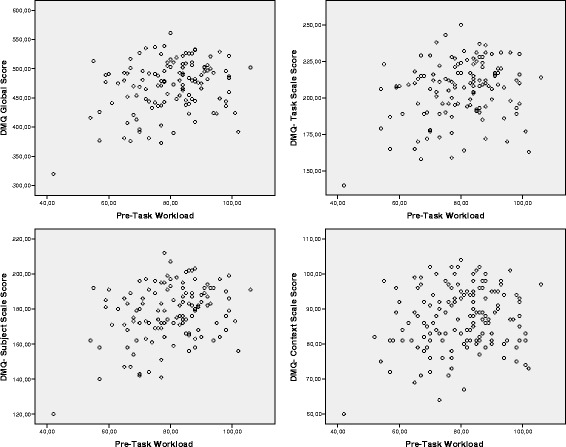
Scatter plots. DMQ (Decision-Making Questionnaire) vs. Pre-Task Workload. *DMQ* Decision-Making Questionnaire

As seen in Table 2, our data show evidence of a positive and moderate linear-relation-expected mental workload (Pre-Task WL) and the different scales and subscales of the DMQ. However, as the existing research shows potentially nonlinear relationships between MWL measures and performance, we explored the general pattern of relationships between the DMQ measures and Pre-Task WL scores by means of scatter plots. The examination of these plots, which are presented in Fig. 1, revealed that medium values of Pre-Task WL are potentially associated with higher DMQ scores when compared with high and low Pre-Task WL values.

In order to tentatively explore this pattern, we divided Pre-Task WL scores into three groups, taking as criteria the values of the mean (*M* = 80.3) and standard deviation (SD = 12.3). Consequently, three groups were defined: (1) group with Low Pre-Task WL (lowest value to *M* − SD); (2) group with Medium Pre-Task WL (Mean − SD to Mean + SD); (3) group with High Pre-Task WL (Mean + SD to highest value). We used MANOVA to contrast the differences in the quality of decision-making among the three groups. DMQ-Total, DMQ-Task, DMQ-Subject, and DMQ-Context scores were included as dependent variables and Grouped Pre-Task WL was included as factor.

When testing the parametric assumptions, it was observed that the variables referring to decision-making did not follow a normal distribution. The four chosen indicators (DMQ-Total, DMQ-Task, DMQ-Subject, and DMQ-Context) obtained values in the Kolmogorov-Smirnov test that lead to rejecting the null hypothesis of adaptation to the normal distribution. However, we considered the application of MANOVA to be adequate, given its robustness versus the deviations from normality in symmetrical distributions (Kariya & Sinha, 2014), as is the case of our study. On another hand, the contrast indicators of variance equality of the error term between the groups are satisfactory for all variables. Fit tests were also satisfactory in all cases.

Multivariate tests, which contrast the null hypothesis of the global relation between the different dependent variables and the term of the model, revealed the existence of verifiable relations between the series of the indicators of the DMQ and Pre-Task WL (Wilks’ lambda .853; *F* = 3.382; df 6.246; *p* = .003). Table 3 presents a summary of the inter-subject tests, showing the different indicators that contrast the null hypothesis of a relation between Pre-Task WL and the different indicators of DM.

**Table 3:** MANOVA including DMQ-Total, DMQ-Subject, DMQ-Task, DMQ-Context, and Pre-Task Workload. Inter-subject tests

Dependent variable	*F*	*η* ^2^	Degrees of freedom	Significance
DMQ-Global scale	3.80	.057	2	.025
DMQ-Task subscale	4.07	.061	2	.019
DMQ-Subject subscale	4.44	.066	2	.014
DMQ-Context subscale	2.02	.031	2	.137

*DMQ* Decision-Making Questionnaire

**Fig. 2:**
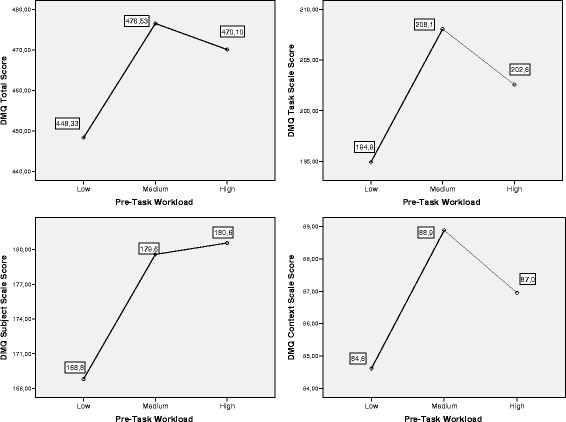
Estimated marginal means. DMQ-Global scale, DMQ-Task subscale, DMQ-Subject subscale, and DMQ-Context subscale vs. Pre-Task Workload. *DMQ* Decision-Making Questionnaire

Relationships are also summarized in Fig. 2, in which estimated means of DM indicators are represented for Low, Medium, and High Pre-Task WL groups. The results revealed the existence of specific and verifiable relations between Pre-Task WL and the DMQ-Total, DMQ-Subject, and DMQ-Task scales. The DMQ-Context scales did not achieve the required significance level. DMQ-Total and DMQ-Task relations with Pre-Task WL showed a similar pattern, in which DM quality seems to be better in the Medium Pre-Task group, slightly lower in the High Pre-Task WL group, and lower in the Low Pre-Task WL group. Scheffé post hoc contrasts (*p* < .05) only yielded significant differences between subjects with Low Pre-Task WL, on the one hand, and Medium and High Pre-Task WL, on the other, for DMQ-Total and DMQ-Task scores. DMQ-Subject, however, showed a different pattern in which subjects included in High Pre-Task WL obtained the highest scores. However, post hoc contrast (*p* < .05) also yielded relevant differences only between Low Pre-Task WL, on the one hand, and Medium and High Pre-Task WL, on the other.

### Relation between Post-Task WL and decision-making

Examination of the correlation matrix reveals low linear correlations between Post-Task WL and the different indicators of decision-making, which only reach significance in a few scales (DMQ-Uncertainty and DMQ-Social Pressure of those scales with adequate reliability). These indicators offer little basis for the exploration of conjoint linear relations. As done for Pre-Task WL, in order to explore potential nonlinear relationships, scatter plots were generated between DMQ indicators and Post-Task WL. However, none of these plots revealed relevant relationship patterns. Thus, low correlations are not justified by the existence of nonlinear relationships, which may concentrate higher values of DMQ scores around medium values of Post-Task WL. On the contrary, they seem to be a consequence of a wide dispersion of DMQ scores over the different values of Post-Task WL.

In order to explore whether the relationships between Post-Task WL and DMQ scores could be concealed by the influence of Pre-Task WL levels, we explored the potential moderation effect of Pre-Task WL in the relationship between Post-Task WL and DMQ. We followed Baron and Kenny’s (1986) indications and performed separate linear regression analysis for Low, Medium, and High Pre-Task WL groups (as defined in the previous section), including Total DMQ scales, DMQ-Task, DMQ-Subject, and DMQ-Context in each case as dependent variables and Post-Task WL as the independent variable. None of the obtained unstandardized regression coefficients was significant. Thus, the moderation effect of Pre-Task WL does not reveal any relevant relation between Post-Task WL and DM.

### Relation between Differential WL and decision-making

**Fig. 3:**
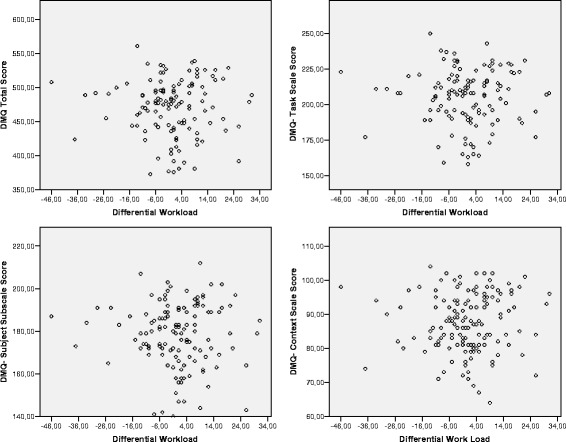
Scatter plots. DMQ vs. Differential Workload. *DMQ* Decision-Making Questionnaire

Table 2 shows the practical absence of linear relation between Differential WL and the DMQ, with linear correlation indices very close to zero in all cases. However, as previously mentioned, we decided to explore potential nonlinear relationships between the two variables. Figure 3 presents scatter plots referring to the Total DMQ scale and the DMQ-Task, DMQ-Subject, and DMQ-Context scales. The scatter plots are compatible with a nonlinear pattern of relation: the levels of Differential WL located around the value zero seem to be clearly associated with lower values on the different indicators of DM. This pattern, which is revealed empirically, has potential theoretical significance because the value zero in Differential WL reflects situations in which there are no differences between expectations of MWL and real MWL, which represents a qualitatively different situation from other situations, from a conceptual viewpoint. To test the consistency of this pattern of relations, we decided to carry out a grouping process of the variable Differential WL, differentiating the group of subjects situated around value zero from subjects with higher and lower values than zero. Among the possible criteria, we decided to delimit the value of percentile 33.33 of the distribution of Differential WL—which was the value −3—and use it as a cutting interval to distinguish three groups: (1) group with Low Differential WL (<−3), (2) group with Adjusted Differential WL (−3 to +3), and (3) group with High Differential WL (>3).

We used MANOVA to contrast the differences in the quality of decision-making among the three groups. On the one hand, we included as dependent variables both the global DMQ score and the score of the DMQ-Task, DMQ-Subject, and DMQ-Context scales. At the same time, given the relations found between Pre-Task WL and decision-making, we decided to include as factors both the grouped Differential WL and Pre-Task WL in order to determine whether the relations between Differential WL and DM may be moderated by the levels of expected mental workload before the task. As the cut point criterion of the two groups of Pre-Task WL, we used the value of the mean (80.26), creating two groups: Low Pre-Task WL (≤80.26) and High Pre-Task (>80.26).

As it was mentioned previously, variables referring to decision-making did not follow a normal distribution, but we considered the application of MANOVA to be adequate, given its robustness versus the deviations from normality in symmetrical distributions, as is the case of our study. On another hand, the contrast indicators of variance equality of the error term between the groups are satisfactory, except for the variable of the DMQ-Subject scale, which presents a Levene test with a significance of *p* < .05. Therefore, the results referring to this variable must be interpreted with caution. Fit tests were, however, satisfactory in all cases.

In Table 4, we show a summary of the main results of the multivariate tests, which contrast the null hypothesis of the global relation between the different dependent variables and the different terms of the model. The results reveal the existence of verifiable relations between the series of the indicators of the DMQ and both Differential WL and Pre-Task WL. However, there is no evidence of a global interaction between the two factors.

**Table 4:** MANOVA including DMQ scores, Differential Workload and Pre-Task Workload. Multivariate tests

Model term	Wilks Lambda	*F*	*η* ^2^	Degrees of freedom (hypothesis)	Degrees of freedom (error)	Significance
Differential Workload (low-adjusted-high)	.868	2.882	.068	6	236	.010
Pre-Task Workload (low-high)	.930	2.980	.070	3	118	.034
Interaction Differential WL × Pre-Task Workload	.984	.314	.008	6	236	.929

Table 5 presents a summary of the inter-subject tests, showing the different indicators that contrast the null hypothesis of a relation between the different factors of the model (Differential WL, Pre-Task WL, and their Interaction) and the different indicators of DM. The results reveal the existence of global relations between the three terms of the model (Differential WL, Pre-Task WL, and their interaction) and all the selected indicators of decision-making, except for DMQ-Context. This global relation is revealed in specific and verifiable relations between Differential WL and the DMQ-Total, DMQ-Subject, and DMQ-Task scales. This relation between the Pre-Task WL and these scales is also verifiable. However, when configuring their relation with the indicators of the DMQ, the two factors did not interact with each other.

**Table 5:** MANOVA including DMQ scores, Differential Workload (Grouped), and Pre-Task Workload (Grouped). Inter-subject tests

Origin	Dependent variable	Degrees of freedom	*F*	*η* ^2^	Significance
Corrected global model (Differential WL. Pre-Task WL. Interaction Differential WL × Pre-Task WL)	DMQ-Total score	5	2.567	.097	.030
	DMQ-Task	5	2.355	.089	.045
	DMQ-Subject	5	3.300	.121	.008
	DMQ-Context	5	1.186	.047	.320
Differential WL (low-adjusted-high)	DMQ-Total score	2	4.515	.07	.013
	DMQ-Task	2	4.424	.069	.014
	DMQ-Subject	2	5.238	.08	.007
	DMQ-Context	2	2.202	.035	.115
Pre-Task WL (low-high)	DMQ-Total score	1	4.743	.038	.031
	DMQ-Task	1	3.568	.029	.061
	DMQ-Subject	1	7.002	.055	.009
	DMQ-Context	1	2.237	.018	.137
Differential WL × Pre-Task WL	DMQ-Total score	2	.641	.011	.529
	DMQ-Task	2	.678	.011	.510
	DMQ-Subject	2	.587	.010	.557
	DMQ-Context	2	.414	.007	.662

*DMQ* Decision-Making Questionnaire, *WL* Workload

**Fig. 4:**
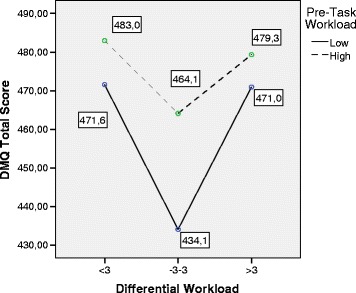
MANOVA. Estimated marginal means. Differential Workload vs. Decision-Making Questionnaire Global score. *DMQ* Decision-Making Questionnaire

**Fig. 5:**
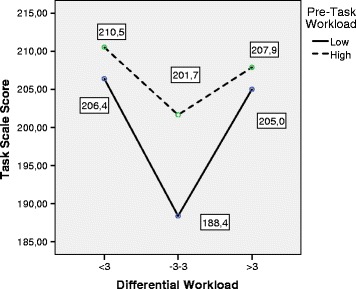
MANOVA. Estimated marginal means. Differential Workload vs. Decision-Making Questionnaire Task scale. *DMQ* Decision-Making Questionnaire

**Fig. 6:**
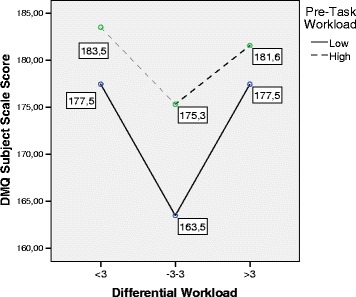
MANOVA. Estimated marginal means. Differential Workload vs. Decision-Making Questionnaire Subjects scale. *DMQ* Decision-Making Questionnaire

**Fig. 7:**
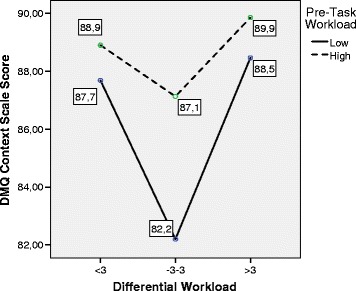
MANOVA. Estimated marginal means. Differential Workload vs. Decision-Making Questionnaire Context scale. *DMQ* Decision-Making Questionnaire

The following graphics, shown in Figs. 4, 5, 6, and 7, specifically summarize the way in which these relations are manifested. The mean values of the different scales of DM are shown as a function of the Differential WL and Pre-Task WL factors. The graphics show that the group with high Pre-Task WL had a greater quality of decision-making (higher scores) in all the indicators than the group with low Pre-Task WL. Only in the case of the DMQ-Context scale did this pattern not reach significance. However, the pattern was substantially different in the case of Differential WL. As shown in the different graphics in a highly consistent way, subjects with adjusted Differential WL showed poorer quality levels of decision-making than subjects with low and high Differential WL. The pairwise post hoc comparisons (adjusted Differential WL, on the one hand, and low and high Differential WL, on the other) showed that subjects with adjusted Differential WL maintained significantly lower scores (*p* < .05) of Total DMQ, DMQ-Task, and DMQ-Subject. The pairwise comparisons also revealed that these subjects obtained lower scores in DMQ-Context than subjects with high Differential WL, although there was no global significance between the two variables. Lastly, there were no testable differences in the indicators of decision-making between subjects with high and low Differential WL in the post hoc tests.

## Discussion

In the present work, we have attempted to deepen our knowledge of the relations between a process of great relevance in complex organizational settings, decision-making (DM), and an indicator of occupational quality with a long trajectory, mental workload (MWL). For this purpose, we used theoretical models and instruments that have been contrasted in prior literature. We analyzed real tasks at the workplace in subjects who carry out their activity in an organization, and we focused on DM from a model that takes into account the particularities of professional settings. The results obtained reveal some consistent and expected evidence, but they also present innovative relations that are necessary to interpret.

The initial analyses of our study reveal the psychometric robustness of the DMQ when used as a global score. It also shows good psychometric indicators for the DMQ-Task and DMQ-Subject subscales and somewhat worse indicators for the DMQ-Context subscale. Different subscales also show adequate reliability indicators (Uncertainty, Time/Money Pressure, Information and Goals, Consequences of Decision, Cognition, and Social Pressure), although there are others, especially those referring to Motivation and Self-Regulation, which show significantly lower psychometric indicators than those obtained in the initial studies that led to the construction of this instrument (Soria-Oliver, 2010). These results reinforce the suitability of the instrument as a whole and also its main dimensions. On another hand, the results point to the need to continue contrasting the application of the subscales and to determine the extent to which—and with different populations—the separate use of the subscales can be maintained.

From the viewpoint of the descriptive statistics, some reflections are warranted. A notable aspect of our study is the obtention of indicators in a professional sample that, in turn, is representative of a specific organization. This type of samples is scarce in the literature (Díaz et al., 2010), which has preferentially focused on laboratory tasks and student samples. On another hand, there are few studies that report data from the chosen approach, which suppresses the weighting phase of the NASA-TLX scale. From this perspective, it is not possible to establish comparisons with the absolute score when assessing the significance of the scores of our sample. Nevertheless, if we take into account that the mean levels of the measurement scale as a whole reach 60 points and that the maximum is 120 points, our sample is located around the medium-high level in absolute terms. Regarding decision-making, we can use previous works carried out with different professional groups as a reference (Sanz de Acedo Lizarraga et al., 2009). Based on them, it should be noted that our sample presents higher values of quality of DM than the samples used in the same geographic context.

From the viewpoint of exploring the relations among the different variables, we found different patterns as a function of the moment and the way of implementing the MWL measure.

In this sense, with regard to the first goal of this study, which was to explore the relation between the expected MWL, which we have called Pre-Task WL, and the quality of DM, results provide support for our hypothesis. Thus, from a global point of view, the relationship between Pre-Task WL and DM is compatible with the expected pattern proposed on the basis of the existing literature (Brookhuis & Waard, 2002; Wilson & Rajan, 1995; Young & Stanton, 2002; Young et al., 2015). Specifically, global DM as measured by the Total DMQ score and DM aspects related to the way in which the task is handled yielded better quality when intermediate levels of MWL were anticipated. Our results are concordant with those presented by Wilson and Rajan (1995) and Young and Stanton (2002) in the case of operative tasks and by Jackson et al. (2014) specifically for DM, pointing out that both under- and overload could lead to poorer performance. In this sense, on the one hand, underload may lead to inadequate activation and scarce use of workers’ available resources. Our results are also perfectly compatible with evidence indicating that an adequate process of activation is useful for better performance in cognitive tasks both in academic contexts (Pekrun, Goetz, Daniels, Stupnisky, & Perry, 2010) and organizational settings (Fisher, 1993; Loukidou, Loan-Clarke, & Daniels, 2009). However, a slightly different pattern was found for the relationship between expected MWL and the performance of DM strategies related to the subject dimension. In this sense, although DM performance referring to the subject dimension increases when expected MWL remains at a medium level, the quality of these strategies decreases when expected MWL is higher. This fact, taken with caution, indicates that subjects’ ability to engage in the decision process in a motivated, thoughtful way and by self-regulating the process may not be affected, but maintained or improved when higher MWL is expected. This result may be explained by the fact that DM strategies referred to the subject dimension are more accessible to self-regulation than DM strategies that affect task or environment handling (Cannon-Bowers & Salas, 2002).

Taking into account the above and, in any case, considering the study’s limitations, our study provides evidence for the application of results to DM in organizational contexts. In this sense, our results indicate that anticipating levels of MWL that guarantee workers’ adequate activation may yield better results in DM performance. The expectation of low task requirements may lead to workers’ poorer quality of decisions. High levels of expected MWL could lead to a decrease in DM performance, but this decrease seems to be moderated by the kind of task that has been explored, related mainly to intellectual work, and there is no evidence for the dimensions related to subjects’ motivation and self-regulation.

With regard to the second goal: to explore the relation between Post-Task WL and DM, the results do not yield evidence of a relation between the two constructs. Different kinds of potential relationships were explored (linear and nonlinear), as was the potential moderation effect of Pre-Task WL, but none of our analyses showed relevant patterns. This indicates that, from our data, we cannot make direct inferences about the quality of DM that the worker performs as a function of the MWL experienced. This finding is not consistent with our hypothesis, which assumed that an “inverted U” pattern would be found between DM and real experienced MWL. Our hypothesis, as previously mentioned, was based on the existing knowledge about the general relation between performance and MWL (Brookhuis & Waard, 2002; Wilson & Rajan, 1995; Young & Stanton, 2002; Young et al., 2015) and some specific results referring to relationships between MWL and DM (Jackson et al., 2014). Some possible mechanisms could explain this lack of relation: (1) Our results could be conditioned by the levels of MWL present in the studied sample, which may not reflect the values of low or high MWL achieved by experimental manipulation in common simulation studies (Young et al., 2015). Thus, our results could be revealing a comfort zone, in which the variations in MWL scores do not reflect an important change in conditions affecting DM performance. However, this condition may not operate in the same way for expected MWL. In this sense, as shown previously, expected MWL—although yielding a similar distribution—showed relevant effects on DM. (2) Combined with this, experienced MWL in real organizational settings may be handled dynamically (Weick & Sutcliffe, 2008) by means of different strategies (e.g., rebalancing demands and capabilities) that may reduce activation level and, consequently, MWL impact on DM. In this sense, DM in natural settings may offer subjects more flexibility to buffer the effect of MWL than do closed experimental or operative tasks. (3) Additional variables, like subjects’ expertise or stress reaction and appraisal, could be operating as mediators between MWL and DM (Weick & Sutcliffe, 2008; Starcke & Brand, 2012).

In relation to our third goal, the exploration of the relations between decision-making and the differential mental workload, the pattern of relations found is striking and of great interest. However, it differed partially from our expectations according to our hypothesis. In this sense, we found that the relationships between Differential WL and DM are not moderated by expected WL (Pre-Task WL), as Differential WL and Pre-Task WL had no significant interaction and presented parallel relationship patterns with DM. Additionally, the results show that the workers whose expectations of MWL match the real MWL would have a worse quality of DM than those whose MWL is higher or lower than expected. The relation occurs both for global DM and the processes that affect the task and the subject, which are reflected to a lesser extent in the dimensions of decision-making that affect the setting. This pattern of results leads to considering that when there is a match between expectations and the real task, the task demands do not activate a better quality process of DM. According to our theoretical basis, this result should be explained by the fact that this match may not place subjects in the optimum activation range but in a potential underload zone that may lower DM performance (Wilson & Rajan, 1995; Young & Stanton, 2002; Jackson et al., 2014). The higher quality in DM in the group with more MWL than expected is also concordant with our hypothesis and may be revealing a higher cognitive demand that places subjects on a better performance range and, on another hand, does not exceed the limits to generate a deficient performance. This pattern has been previously shown in MWL for general performance (Young & Stanton, 2002; Young et al., 2015) and DM (Jackson et al., 2014). The presence of higher levels of DM quality in the group with lower MWL than expected is more difficult to explain because it is not consistent with the existing evidence from other studies (Jackson et al., 2014; Loukidou et al., 2009). This could be revealing the possibility of developing more qualified DM in the absence of task demands, but we have no basis to contrast this hypothesis with our data. In any event, it seems to reveal the need to consider dynamic and more complex processes, such as the reciprocal influence between the two constructs. The exploration and determination of these patterns of relation would be of great interest to adequately design the levels of MWL that allow more qualified organizational decisions.

One strength of our study is that we measured MWL before and after the task, as well as the fact that it was carried out in a real professional setting. However, it has the limitation of not ensuring more extreme values of MWL, which would allow a better contrast of their effect on DM. Such values could be obtained by including some quasi-experimental manipulation or by accessing settings regularly submitted to higher mental loads. This would allow comparing the extent to which the patterns found are repeated, especially for the complex relations found between DM and differential WL. It may also be of interest to complement the quantitative indicators with a qualitative follow-up of the process undergone by the subjects, in order to further our understanding of the unexpected results we found. Our study design may also be improved if potential moderators of the relationship between MWL and DM are considered. In this sense, subjects’ expertise could be one of the most relevant factors to be included in further studies.

## Conclusions

Our work reveals the existence of complex relations between the MWL experienced by workers in a setting with intellectual tasks and the quality of the DM they develop in their work. Quality of DM appears to be better when medium levels of WL are expected. Underload or overload may affect general quality of DM and, specifically, the strategies related to task management. However, decision strategies related to the subject’s own motivation and self-regulation appear to be less affected by overload. This relationship pattern shows the potential positive effects of adequate activation prior to the task on DM performance. However, this effect does not seem to be linked to the real MWL experienced because we found no consistent pattern of relation between the quality of DM and the MWL experienced after having carried out the task. The relation between the differential WL, on the one hand, and the quality of DM, on the other, showed also a nonlinear pattern. Thus, the workers whose expected MWL matches the one they must face have a worse quality of DM than those who have a mismatch between the two, either in a positive or negative sense. This finding makes us consider a more elaborate pattern of relations, in which the possibility of the existence of a reciprocal dynamic influence between the two constructs should be contemplated. Our results lead to various questions, which can be taken up by future research to better understand the influence of the levels of overload in complex tasks that are dealt with in current organizational settings.

## Abbreviations

DM: Decision-making
DMQ: Decision-Making Questionnaire
MWL: Mental Workload
NASA-TLX: National Aeronautics and Space Administration-Task Load Index
WL: Workload
